# BioKnit: development of mycelium paste for use with permanent textile formwork

**DOI:** 10.3389/fbioe.2023.1229693

**Published:** 2023-07-14

**Authors:** Romy Kaiser, Ben Bridgens, Elise Elsacker, Jane Scott

**Affiliations:** ^1^ Hub for Biotechnology in the Built Environment, School of Architecture, Planning and Landscape, Newcastle University, Newcastle upon Tyne, United Kingdom; ^2^ Department of Bioengineering Sciences, Research Group of Microbiology, Vrije Universiteit Brussel, Brussels, Belgium

**Keywords:** mycelium, textile formwork, knit, biofabrication, mechanical properties, mycocrete paste

## Abstract

This paper presents significant advances in mycelium biofabrication using permanent knitted textile formwork and a new substrate formulation to dramatically improve the mechanical properties of mycelium-textile biocomposites suitable for large-scale components for use in construction. The paper outlines the biofabrication process, detailing the composition of *mycocrete*, a viscous mycelium substrate developed for use with permanent knitted formwork, and the injection process required to regulate the filling of slender tubes of fabric with mycocrete. The use of a permanent integrated knitted formwork shows promise as a composite system for use with mycelium to improve mechanical performance and enable complex shapes to be fabricated for lightweight construction. Results of mechanical testing show dramatic improvements in tensile, compressive and flexural strength and stiffness compared to conventional mycelium composites. The testing demonstrates the importance of both the mycocrete paste recipe and the knitted textile formwork. In addition, the paper highlights the advantages of the proposed biofabrication system with reference to the *BioKnit* prototype: a 1.8 m high freestanding arched dome composed of very slender biohybrid knit-mycelium tubes. This prototype demonstrates the opportunity to utilize the potential for lightweight construction and complex form offered by a textile formwork with low environmental impact mycelium biomaterials. The combination of textiles and mycelium present a compelling new class of textile biohybrid composite materials for new applications within the construction sector.

## 1 Introduction

The urgent need to address the climate impact of the construction industry has catalyzed interdisciplinary researchers to explore sustainable alternatives of traditional building materials and construction methods. Over the last 10 years there has been a huge expansion in research focused on biomaterials and specifically composite materials biofabricated from mycelium (the root network of fungus) and bio-based substrates such as sawdust or straw (Elsacker et al., 2020). The fabrication of mycelium composites can be a low energy and carbon neutral process (Jones et al., 2020), using agricultural by-products as both bulk aggregate and nutrition for the mycelium that can be grown at temperatures around 25°C. Mycelium composites have huge potential as a material for construction because they demonstrate excellent thermal and acoustic properties (Jones et al., 2020) and can therefore be used as both insulation and soundproofing. They have potential to provide an inexpensive and sustainable class of materials suitable for the replacement of foams, timber and plastics for applications within building interiors (Jones et al., 2020). Mycelium composites have already found commercial applications in packaging (Ecovative, 2023) and insulation panels (Mogu, 2023). Alongside commercial products researchers have developed larger bespoke architectural structures such as MycoTree (Heisel et al., 2017), the Growing Pavilion (Pascal Leboucq in collaboration with Krown Design) and the HyFi Tower composed of 10,000 bricks and exhibited at the Museum of Modern Art in New York in 2014 (Benjamin, 2017). However, due to the novelty of the material a gap exists between the vision for “growing a building” (Dade-Robertson, 2020) and the development of both the constituent materials and the biofabrication processes suitable for large-scale implementation in architecture and construction, with specific challenges regarding complexity in shape, structure, stability, and scalability.

### 1.1 Biofabrication of mycelium composites

Conventional mycelium composites are grown in a rigid mold. Mycelium spawn (a mixture of growing mycelium spores and a nutrition source such as millet or rye) are mixed with a substrate material such as sawdust or straw (Elsacker et al., 2019). Substrate materials are sterilised prior to inoculation with mycelium to prevent contamination from other microorganisms during growth. The mycelium composite mixture is tightly packed into the mold, and placed in controlled environmental—with high humidity, darkness, and temperatures around 25°C for 7–14 days. As the mycelium grows, it colonises the substrate binding the materials together in a web of entangled hyphae (the individual mycelium roots). The mycelium composite takes the form of the mold in which it is grown. After the growth stage, and prior to the growth of fruiting bodies (mushrooms), the mycelium is rendered inactive, either through heat treatment in an oven (Elsacker et al., 2019; Elsacker et al., 2020), or through air drying (Agraviador et al., 2022).

The resultant mechanical properties achieved in mycelium composites depend on a wide variety of factors; fungal species, substrate composition, the interaction of species and substrate, the growing conditions (light, temperature and humidity), as well as post-growth treatments such as the method to render the mycelium inactive and any physical processing (e.g., heat pressing) or chemical processing (Appels et al., 2019). The physical and mechanical properties of mycelium composites vary dependent on the substrate on which it is grown; this varies from composites with a density in the range of 60–130 kg/m3 for straw based substrates, to a higher density of 87–300 kg/m3 for sawdust based substrates (Jones et al., 2020). Use of sawdust as the substrate generally results in higher strength and stiffness values (Jones et al., 2020). The tensile strength of mycelium composites varies according to different studies, with values of 0.05 and 0.18 MPa reported for sawdust based composites, with elastic moduli of 1.30 and 13.0 MPa. In compression, strength values of 0.49 and 1.1 MPa are reported, with elastic moduli of 0.14 and 1.0 MPa. In flexure, strength reported in three studies varies from 0.05 to 0.29 MPa, with elastic moduli between 1.0 and 9.0 MPa (Jones et al., 2020). To achieve good mechanical properties in the mycelium composite the interaction between mycelium and substrate is critical because the density of the hyphae network is dependent on the ability for mycelium to access high quality nutrition from the substrate (Jones et al., 2020).

The majority of biofabrication strategies are limited to bricks and blocks using rigid plastic molds as described above. These are designed to be assembled using traditional construction techniques, for example, as blockwork (Benjamin, 2017) or used as cladding (The Growing Pavilion, 2023). One challenge for this kind of mycelium biofabrication is that size and volume limitations for the growth of mycelium which limit the thickness of a mycelium composite to about 150 mm because of the organism’s need for oxygen in order to grow (Elsacker et al., 2020). From a design perspective, the formation of mycelium bricks limits the transformative opportunity for biofabrication to explore complex shapes and lightweight, efficient geometries with the potential to disrupt the building process and develop new forms of construction. More recent approaches look to digital fabrication strategies as 3D printing or textile-based formwork for mycelium composite biofabrication. These approaches aim to create more complex forms, and work more sympathetically with the growth of the organism and require more sophisticated substrates and application processes tailored to production.

### 1.2 3D printing and extrusion-based techniques

The opportunity to 3D print inoculated growth substrate has been explored by several groups (Colmo and Ayres, 2020; Goidea et al., 2020; Soh et al., 2020; Bhardwaj et al., 2021; Blast Studio, 2021; Elsacker et al., 2022; Bio Ex Machina, 2023). The challenge in this approach is to develop an extrudable but stable substrate mix consistency, that is, viscous enough to allow extrusion through a 3D print nozzle, but stiff enough to be self-supporting prior to mycelium growth (Elsacker et al., 2022). Therefore particle size, pressure and movement of robotic extrusion mechanism are critical design criteria (Elsacker et al., 2022). To achieve the correct viscosity for extrusion the addition of gelling agents has been explored. In research by Elsacker et al (2022), the addition of 5%–15% gelling agent (e.g., psyllium husk, locust bean gum, xanthan gum, guar gum and paper cellulose) within the substrate mix does not impact mycelial growth. A range of additives identified in the work by Elsacker (ibid) form the basis for the development of a suitable paste consistency in the research detailed in this paper.

### 1.3 Textile formwork for mycelium composites

The use of textiles to provide formwork for mycelium-based composites has been explored by different groups looking for solutions to produce lightweight, self-supporting mycelium structures using efficient geometries and complex shape. Examples include woven structures such as 3D Kagome weaving processes (Adamatzky et al., 2021), 3D knitting (Yogiaman et al., 2020; Scott et al., 2021) and fabric formwork (Dessi-Olive, 2022). In each of these examples the textiles provide a soft mold that transitions from flexible to stiff after mycelium growth. Of these approaches, 3D knitting—which has the ability to modify structure, shape and form within one fabric—is a particularly versatile production system. Knitted fabrics have been produced in shaped tubes and tubular branching structures to produce for use as mycelium molds. However, there are challenges in using knit for filled tubular formwork. Firstly, knitted fabric is extensible because the fabric is composed of intersecting loops of yarn. For use with mycelium, this can lead to stretching and distortion during filling (Scott et al., 2022) which compromises the strength of the resulting composite. In addition, when working with large tubular knitted components it becomes difficult to fill the tubes with the mycelium mix as the fabric stretches. The conventional method is to “hand pack” a mycelium mold, manually pushing handfuls of mycelium substrate into the knitted fabric tubes. However, hand packing soft molds can produce an uneven fill leading to poor growth (Beyer, 2019), potential contamination (Biala and Ostermann, 2022) and poor mechanical strength, resulting in components that fail to be self-supporting (Beyer, 2019; Biala and Ostermann, 2022; Scott et al., 2022).

### 1.4 Introducing BioKnit

**FIGURE 1:**
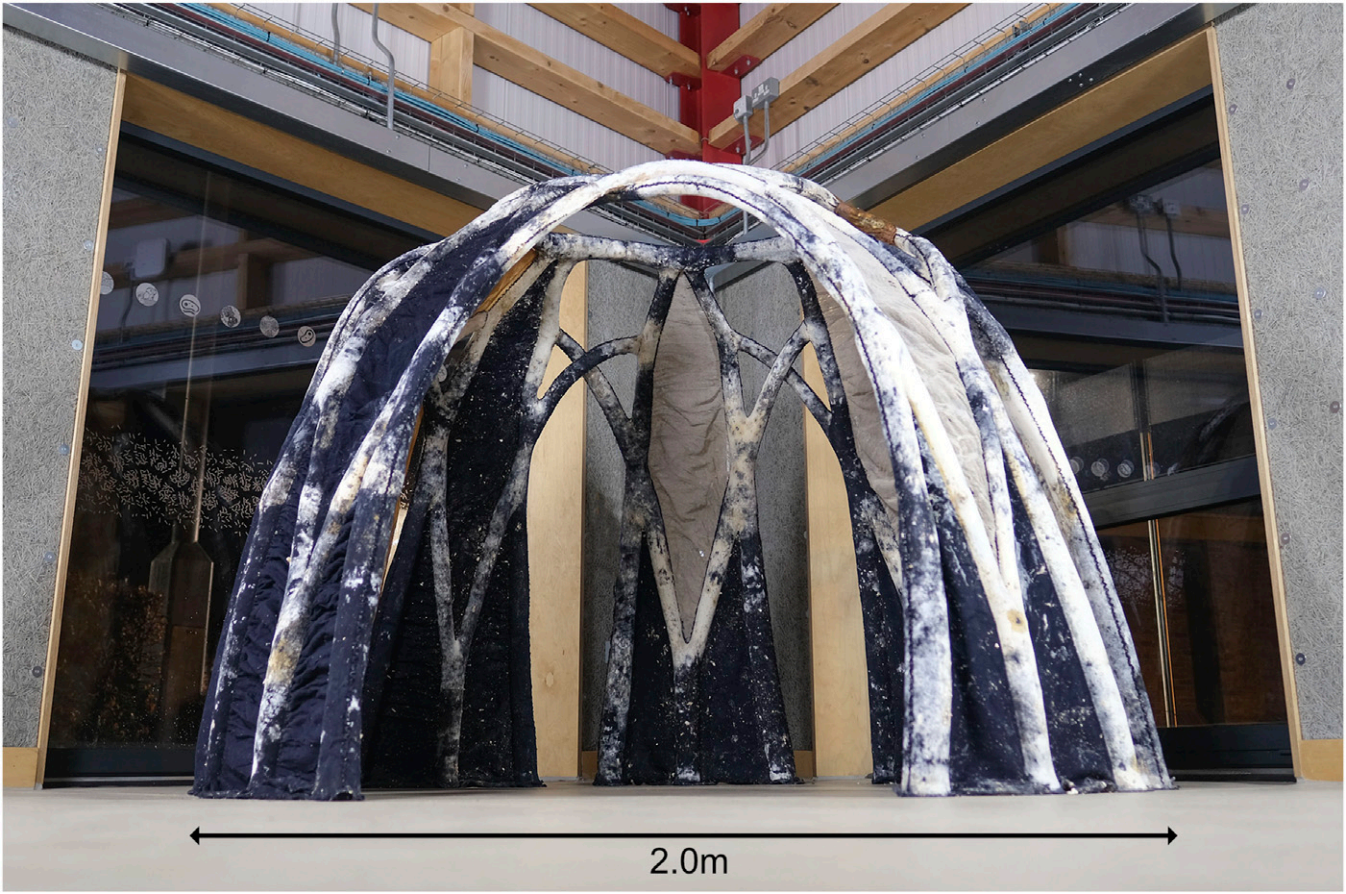
BioKnit self-supporting structure exhibited in the OME, Newcastle University (Image credit: Hub for Biotechnology in the Built Environment).

BioKnit was created in 2022 by the Living Textiles Research Group led by Jane Scott, part of the Hub for Biotechnology in the Built Environment (www.bbe.ac.uk), and demonstrated how knitted fabric could be used as formwork for mycelium growth in the biofabrication of a 1.8 m high, 2 m diameter freestanding structure (Agraviador et al., 2022; Scott et al., 2022). BioKnit was composed of seven 3D modules knitted on a 12 g g Shima Seiki SSR industrial knitting machine. The modules were assembled by hand into a single preform, creating permanent textile formwork for a mycelial architecture (Figure 1).

In the development of the prototype a series of biofabrication protocols were established for textile biohybrids. The pre-tests (Scott et al., 2022), form finding and the introduction of a custom-made environmental chamber (Agraviador et al., 2022) are discussed elsewhere. This paper addresses specific challenges of working with soft, extensible knitted fabrics by designing: 1) a mycelium substrate mixture, and 2) a production system suitable for the application of mycelium substrate mixture with permanent knitted formwork. The paper details the formulation of the recipe for mycocrete, a specialised mycelium paste designed to work with textile formwork. Results show that the mechanical strength of the composite is improved by the combination of knitted fabric formwork and the mycocrete mixture. In addition, the paper discusses the application of mycocrete using an injection system using an injection gun to produce a standard, even fill when working with a viscous paste. The success of the system for large scale applications is discussed with reference to BioKnit.

## 2 Materials and methods

### 2.1 Experimental strategy

The aim of the tests was to determine the mechanical properties of the proposed mycrocrete mix within a permanent textile formwork, and compare those properties to mycocrete without textile formwork or without mycelium, to elucidate the impact of the textile formwork and the mycelium on the mechanical properties of the BioKnit material. In addition, testing was carried out on a typical mycelium material with beechwood sawdust as the substrate for comparison with the new material. Four different sets of material samples were therefore required (Table 1).

**TABLE 1:** Experimental strategy.

Sample number	Substrate composition	Inoculated with mycelium?	Enclosed in knitted textile formwork	Aim of experiment
1	Beechwood sawdust	Yes	Yes	Enable comparison of mycocrete mechanical properties compared to a typical mycelium material
2	Mycocrete paste	Yes	Yes	Assess properties of proposed BioKnit material, i.e., mycocrete paste within knitted formwork
3	Mycocrete paste	Yes	No	Assess the impact of the knitted formwork on mechanical behaviour
4	Mycocrete paste	No	Yes	Assess the impact of the mycelium on mechanical behaviour

### 2.2 Cultivation of mycelium and sawdust

Moist beechwood sawdust was prepared with 50% wood and 50% water (by weight) and filled in autoclavable bags and sterilized at 121°C for 30 min. After cooling, the beechwood fibres were inoculated with 10% *Ganoderma lucidum* (Strain M9726, purchased from Urban Farm-It, United Kingdom), with all equipment sterilised with ethanol and a Bunsen burner flame nearby to minimise the risk of contamination. The material was stored in plastic autoclavable growth bags with an air filter by the company. The substrate was then placed in a dark growth chamber at 26°C–27°C for 4 days. On days 2 and 3 the bags were turned and mixed by manipulating the outside of the bags by hand, to ensure evenly distributed growth and oxygen distribution. After 4 days the mycelium growth was paused for 19 days by placing the bags of sawdust and mycelium in a fridge at 5°C as the lab and growing room was required for another project. This was the same for all samples tested. Following this pause, the mycelium and sawdust was placed in the growth chamber for 2 days to revive the mycelium before the samples were prepared.

### 2.3 Standard mycelium material

The standard mycelium material (sample type 1) consisted of moist beechwood sawdust inoculated with mycelium as described above.

### 2.4 Mycocrete paste

The mycocrete paste used in samples types 2, 3 and 4 consisted of beechwood sawdust (10.2%) soaked with water (10.2%) and inoculated with mycelium spawn (2%) described above under *Cultivation of mycelium and sawdust*, mixed with the additives paper powder (4.6%), paper fibre clumps (4.6%), water (65.6%), glycerin (1%) and xanthan gum (1.6%) as listed in Table 2. During preliminary tests proportions of the additives were chosen to provide the correct consistency (Scott et al., 2022). The paste needed to have a low enough viscosity to be injected into the tubular textile formwork, whilst being firm enough to hold its shape and not slump excessively (which could result in excessive deformation or failure of the textile formwork), and dry enough to avoid leakage and allow oxygen to reach the mycelium.

**TABLE 2:** Mycocrete paste.

Material	Details	Proportion by weight used in mycocrete (sample types 2, 3 and 4) (%)	Purpose
Beechwood sawdust	Räuchergold, J.Rettenmaier and Söhne; Rosenheim, Germany	10.2	Standard cellulosic substrate material
Water used to wet sawdust prior to inoculation	Boiled tap water, allowed to cool before use	10.2	Moisture for mycelium growth
Mycelium spawn	*Ganoderma lucidum* (Strain M9726)	2.0	Chosen for rapid, reliable growth
Paper powder	Cellulose Powder (DAS Papier-Mache Powder) by FILAGroup Company, France	4.6	Cellulosic substrate material, nutrition for mycelium, smooth paste
Paper fibre clumps	Unbleached paper bedding from Small Pet Select Limited, United Kingdom	4.6	Cellulosic substrate material, nutrition for mycelium, coarse structure ensures oxygen can reach the mycelium
Water	Boiled tap water, allowed to cool before use	65.6	To achieve injectable paste consistency in combination with gelling agents
Glycerin	Naissance Vegetable Glycerine Liquid No. 806	1.0	Gelling agent to control viscosity
Xanthan gum	Food Supplement E415 Stabiliser Emulsifier Binder	1.6	Gelling agent to control viscosity, also provides nutrition

All materials were autoclaved before mixing, either in autoclavable bags or for glycerol in a glass bottle. All autoclave cycles were 121° for 30 min. The mycocrete paste materials were thoroughly mixed in a sterile container, with all equipment and surfaces sterilised with ethanol.

### 2.5 Textile formwork

The fabric tubes were programmed using a Shima Seiki Apex3 Knitting Machine System and knitted on a 12 g g Shima Seiki SSR122. Tubes were knitted from 2 ends of 2/30 nm merino wool (Uppingham Yarns) at a stitch setting of 70 to create a fabric with stitch density of 56 stitches per cm^2^. The tubes for the rectangular samples measure 36 stitches or 63 mm wide when flat in a relaxed state. The round tubes measure 44 stitches or 54 mm diameter in a relaxed state. The fabric tubes were autoclaved before use by wetting with approximately 25% water by weight of textile and were packed in autoclavable bags for the sterilization process. All autoclave cycles were at 121° for 30 min.

### 2.6 Specimen preparation: filling and growth

Plastic molds were used for all samples to ensure consistent dimensions for mechanical testing. The following description of specimen growth applies to sample types 1, 2, and 3. Sample type 4 does not contain mycelium and therefore was not placed in the growth chamber for incubation. However, the procedures for filling the molds and drying were identical for all sample types.

**FIGURE 2:**
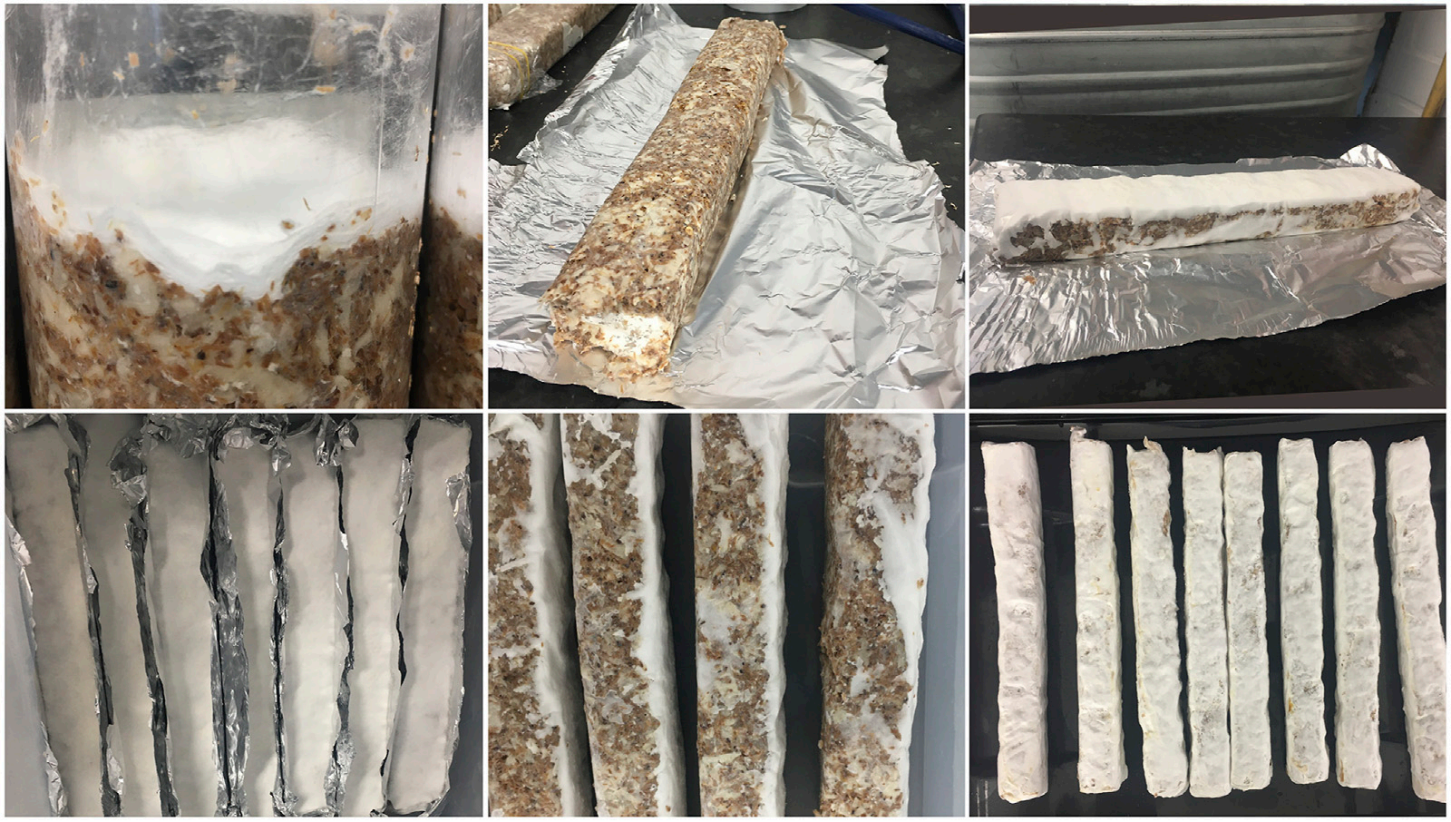
Demolding process and growth of sample type 3 (mycocrete paste without fabric): Growth within plastic mold entirely on surface area (top left); demolded paste sample with low level of mycelium growth (top centre); after second growth stage (top right); samples with aluminum foil supports, top view after second growth stage (bottom left); turned samples (bottom centre); fully grown samples at the beginning of the drying stage (bottom right). Sample size: 260 × 27 × 42 mm.

To prepare the test specimens, a fabric tube was stretched into place inside each mold and fixed with rubber bands and tape on the outside of the mold to maintain the tension in the fabric whilst filling. The molds were filled by hand with mycocrete paste, using a hand tool to ensure even filling. Once the molds were filled they were placed in the growth chamber (dark, 26–27 deg. C, 60%–65% RH) for 8 days. After 8 days the samples were removed from the molds. The samples were pushed out of the molds to enable the mycelium to continue growing with an improved supply of oxygen. The type 3 samples had not grown enough to be self-supporting, and therefore aluminium foil was shaped to provide some support around the specimen, whilst allowing air to reach the specimen on one side (Figure 2). The samples were placed in plastic boxes to ensure high humidity (70%–90% RH) to prevent them drying and were returned to the growth chamber for 5 days, they were then turned and grew for a further 3 days, to try to ensure even growth.

### 2.7 Specimen preparation: drying

Mycelium composite materials are usually oven dried to rapidly remove moisture. For large scale applications such as the BioKnit prototype, which was grown *in situ* in a single piece, oven drying is not feasible (Agraviador et al., 2022). We therefore tried to air dry the test specimens to replicate the planned BioKnit fabrication process. After the growth phase, the samples were removed from the plastic container and left in the growth chamber at a temperature of 26°C–27°C to start the drying process. After 10 days of drying in these conditions the samples felt lighter and stiffer, and it was felt that they were dry.

Testing of sample type 1 in compression confirmed that the samples were dry, but the first compression test on sample type 2 revealed that these samples were still moist inside. The influence of the moist sample can clearly be seen in the test results (see ‘Results’ below). To ensure that all remaining samples were fully dried, they were oven dried at 80°C until no further weight reduction was measured.

### 2.8 Mechanical testing

Each sample type was tested in tension, compression and bending, with five repeats of each test, using an Instron Universal 68TM-50 test machine with a 1 kN load cell, with details of each type of test provided in Table 3. For all tests applied force and crosshead displacement were recorded at 0.02 s intervals, and these readings were averaged to provide one data point per second.

**TABLE 3:** Details of mechanical tests.

Test	Specimen dimensions	Fixture	Displacement rate	Standard
Compression	140 mm long x 70 mm diameter	Flat steel platens	10 mm/min	Cylindrical sample with 2:1 ratio of length:diameter (Elsacker et al., 2019)
Tension	260 mm × 27 mm x 42 mm	Flat, serrated steel clamps; distance between clamps 140 mm at start of test	5 mm/min	Based on BS EN IS*O* 527–4:1997 - Plastics. Determination of tensile properties. Test conditions for isotropic and orthotropic fibre-reinforced plastic composites
Bending	260 mm × 27 mm x 42 mm	3 point bending fixture with a span of 110 mm between the supports	10 mm/min	Based on BS EN ISO 14125:1998 Fibre-reinforced plastic composites. Determination of flexural properties

Note: sample dimensions given are the dimensions of the molds. Due to a combination growth after being removed from the mold, followed by shrinkage during drying, final dimensions varied by approximately ± 5%. Each specimen was measured three times in each direction before testing, and the average of the three values was used for stress calculations for each specimen.

The failure stress was identified manually as the maximum stress before a significant drop in stress or stiffness. The failure point cannot be defined more precisely than this as the failure behaviour of the different specimens varies from a clear break point to very gradual reduction in stiffness, with no clear failure point.

For each sample the Young’s modulus was calculated as the secant modulus, i.e., the gradient of a straight line from the origin to a specified point on the stress-strain curve (Table 4). Two secant moduli were calculated for each sample: 1) from the origin to the failure point, 2) initial secant modulus, from the origin to 50% of the failure strain. The initial secant modulus provides a valuable measure of stiffness because 1) for materials that fail gradually and at high strains, the modulus calculated to the failure point can appear artificially low, and 2) in structural design a safety factor will always be applied to reduce the maximum allowable material stress, such that materials will typically be operating in the lower half of the stress-strain curve, well away from the failure point.

**TABLE 4:** Mechanical properties, mean (standard deviation).

	Compression			Tension			Bending		
Sample type	Failure stress	Elastic modulus	Initial elastic modulus	Failure stress	Elastic modulus	Initial elastic modulus	Failure stress	Elastic modulus	Initial elastic modulus
	(MPa)	(MPa)	(MPa)	(MPa)	(MPa)	(MPa)	(MPa)	(MPa)	(MPa)
1	1.16 (0.2)	2.53 (0.23)	2.41 (0.12)	0.17 (0.02)	6.54 (0.81)	9.67 (1.22)	0.43 (0.08)	5.61 (0.57)	7.91 (1.24)
2	0.97 (0.1)	12.29 (1.43)	10.01 (1.44)	0.52 (0.14)	106.87 (34.75)	153.53 (38.04)	1.75 (0.45)	90.05 (20.07)	94.97 (18.32)
3	0.71 (0.11)	10.58 (3.16)	11.45 (2.47)	0.2 (0.08)	53.6 (23.17)	77.85 (29.04)	0.91 (0.34)	72.29 (17.38)	78.29 (20.89)
4	0.88 (0.09)	9.25 (1.56)	8.26 (1.57)	Slippage at clamps			0.94 (0.48)	36.15 (15.76)	39.47 (18.82)

## 3 Results

### 3.1 Growth observations

**FIGURE 3:**
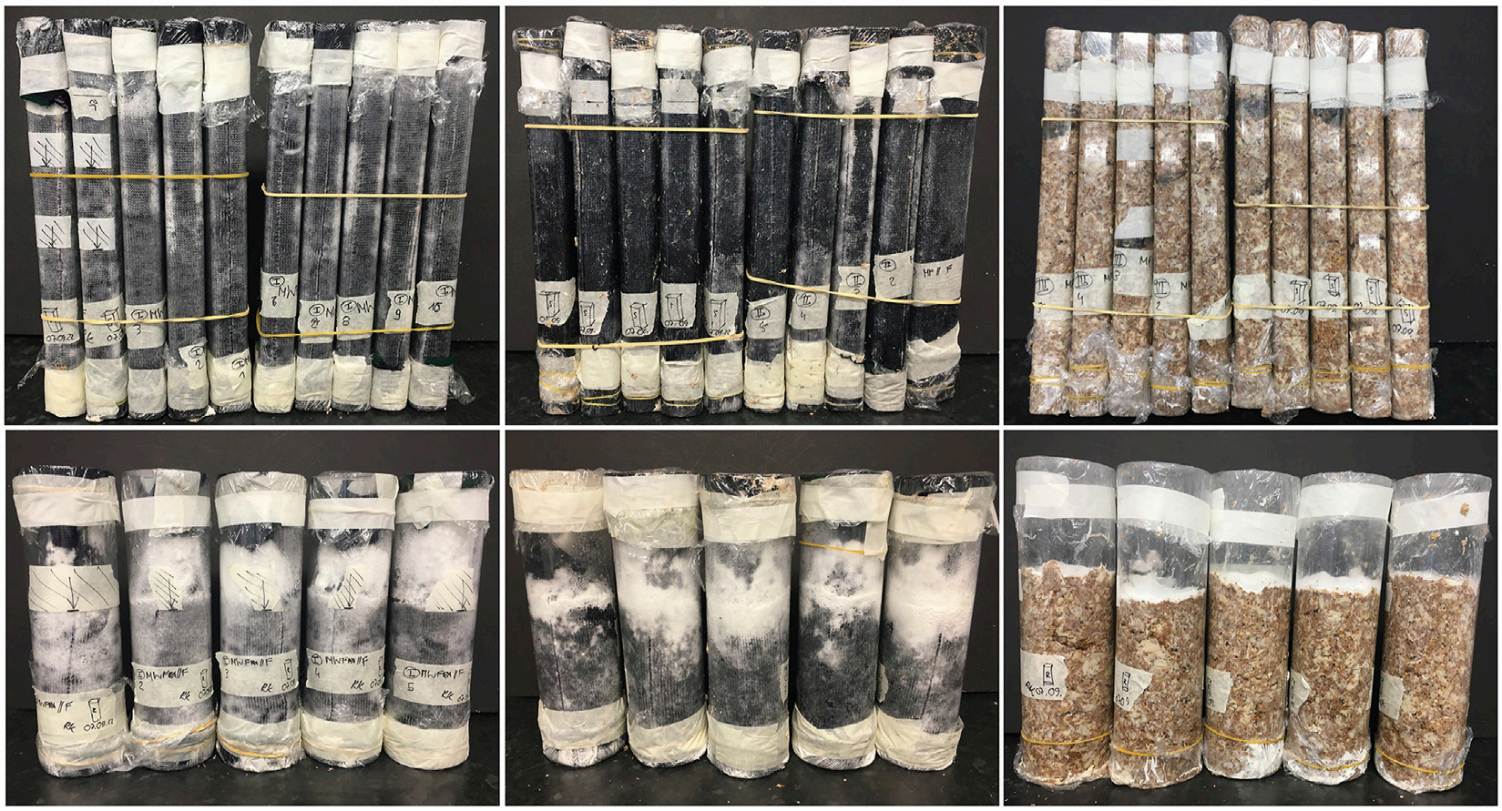
Comparison of growth of sample types 1 (left), 2 (centre) and 3 (right) on day 8 of growth before molds were removed. The top row shows tension and bending specimens (sample size 260 × 27 × 42 mm), the bottom row are for compression testing (sample size 140 × 70 mm). Most growth can be seen on the standard sawdust mix (type 1); with growth also visible on sample type 2. Sample type 3 is lacking visible growth except on the top surface, probably due to lack of oxygen.

The samples within textile formwork tubes (sample types 1 and 2) showed mycelium growth on the outside of the textile formwork, but sample type 3 did not show any visible mycelium growth (Figure 3) during the incubation time in the plastic molds. The mycelium was growing well through the textile surface, especially at the top where there was most oxygen. Growth was even observed at the top of the textile tube, above the level of the substrate filling.

### 3.2 Mechanical test results

**FIGURE 4:**
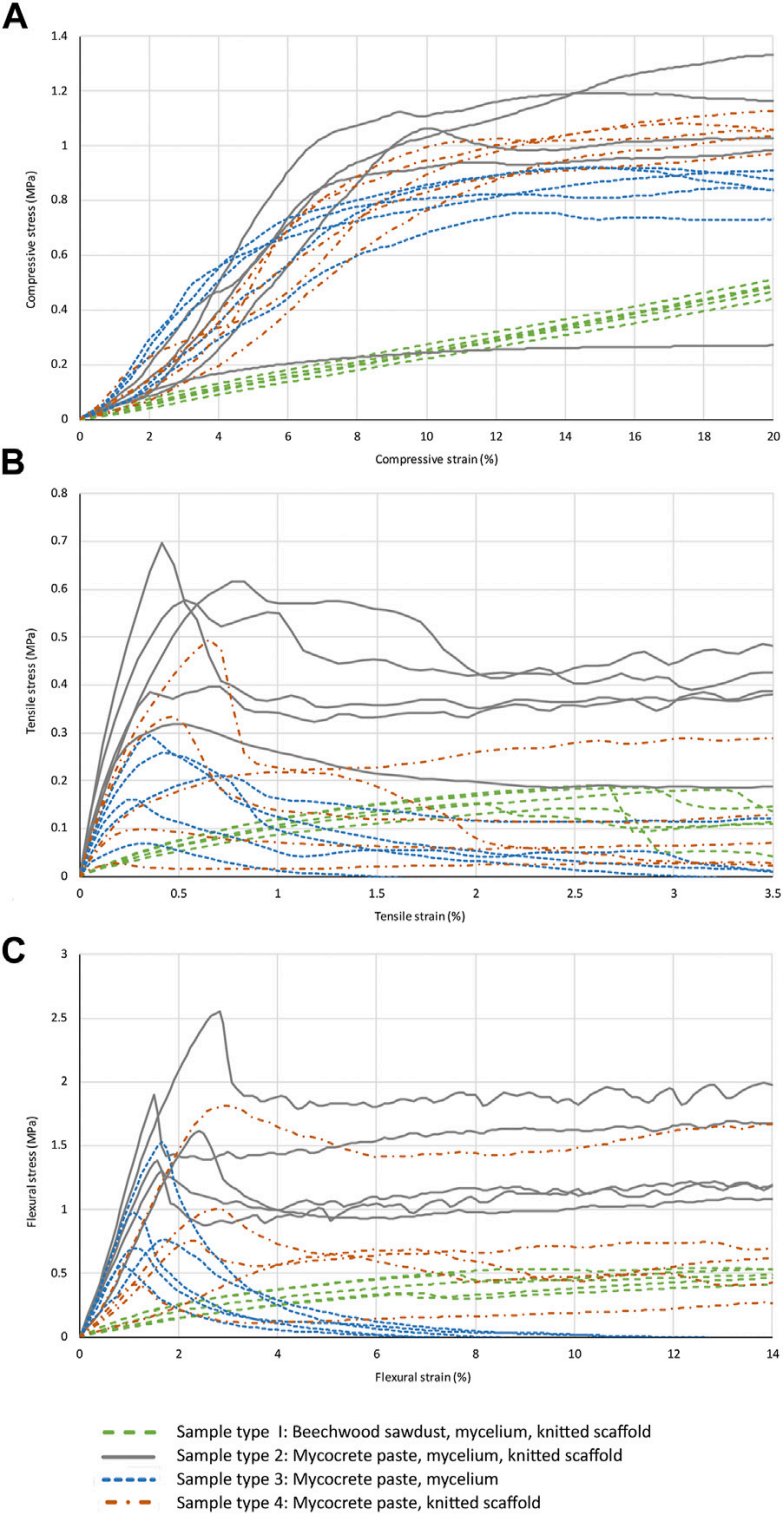
Stress-strain graphs for compression **(A)**, tension **(B)**, and bending **(C)** tests for all sample types.

A complete set of stress-strain curves for all sample types tested in compression, tension and bending is provided in Figures 4A-C, with strength and Young’s modulus values reported in Table 4.

As discussed above (see *Sample preparation: drying*), the first tested specimen of type 2 sample was found to be moist inside after compression testing. The stress-strain curve for this sample can easily be identified in Figure 4A, giving a much lower strength and stiffness than the other four type 2 samples. The curve for this sample has been shown on the graph as it is useful to see the dramatic change in properties when the material is not properly dried, but this sample was not included in the calculation of mechanical properties (Table 4).

Tension testing of relatively weak and/or brittle materials is difficult to carry out reliably as there is a risk that the specimen will either slip out of the clamps if they are not sufficiently tight, or if the clamps are tighter the material may be weakened and the sample will fail at the clamp. The result is low strength values that do not represent the actual material properties, and if gradual slippage occurs at the clamps the modulus will appear to be lower than the true value. For sample type 1, one specimen slipped but from comparison with the four other samples it can be seen that the slippage occurred close to maximum stress, and the stress-strain behaviour up to this point was consistent with the other samples. The sample has therefore been included in the results as a valid test of tensile modulus. For sample types 2 and 3 no slippage occurred at the clamps. For sample type 4 all samples slipped at the clamps at some point in the test. The stress-strain curves have been included in Figure 4B, but no strength or stiffness values have been calculated from this unreliable test data.

## 4 Discussion

### 4.1 Growth observations

The growth of fungal skin growth on the outside of mycelium composites has been associated with improved mechanical properties. It is therefore worth noting that samples 2 and 3 contain higher levels of fungal skin growth (identified as the white coating on samples) compared to 1 and 4. These samples both contain the mycocrete paste mixture. In future work the interaction between the fungal skin and the textile will be analyzed in more detail to ascertain how the mechanical properties of the textiles are impacted through the addition of a fungal skin. During the growth process the textile formwork was positioned between the mycelium/paste mixture and the plastic mold. This porous textile layer increased the oxygen available to the mycelium and contributed to good mycelium growth. Finally, the textile formwork has impacted the way that the composite behaves in the drying stage. Sample 3 which has no textile formwork has reduced in size to a greater extent that the samples with textile formwork. The impact of shrinkage on mycelium composites is important, because up to 75% of the mass during growth is water and so the material can shrink significantly. Further research is required to understand the impact of the textile formwork on the shrinkage rate of mycelium composites.

### 4.2 Mechanical test results

Values of strength and Young’s modulus for sample type 1 (typical sawdust and mycelium composite, within a textile formwork) are similar to those reported in literature (see *Biofabrication of mycelium composites* above), with all values either within the ranges provided in literature, or somewhat higher. This suggests that the mycelium growth and mechanical testing is comparable with other researchers in the field, and that sample type 1 therefore provides a good benchmark against which the mycrocrete and textile formwork results can be compared.

Sample type 2 combines the injectable mycocrete recipe with a textile formwork. The difference between type 2 and type 1 is striking. The mean flexural modulus for type 2 is 90.1 MPa compared to 5.6 MPa for type 1, increasing by a factor of 16. The flexural strength increases by a factor of 4. The compression modulus increases from 2.5 to 12.3 MPa, increasing by a factor of 4.9. Only the compression strength shows a slight reduction for type 2. However, the behaviour of sample type 1 in compression was exceptional—the linear stress-strain curve continued to 40%–50% strain, resulting in relatively high values for compressive strength. Overall, the additives in the mycocrete (paper powder, paper fibre clumps, glycerin and xanthan gum) not only provided an injectable paste, but resulted in a dramatic improvement in mechanical properties. This has been achieved with only a modest increase in density. The average density of the sample type 1 flexural samples was 249 kg/m^3^, and for the type 2 samples it was 306 kg/m^3^, an increase of 23%. Whilst further research is required to fully understand the reasons for this increase in strength and stiffness, possible explanations include 1) the paper powder, paper fibre clumps and xanthan gum all provide an excellent, easily available source of nutrition for the mycelium, and 2) the paper powder and paper fibre clumps will bond the substrate together as they dry even without the mycelium (as tested with sample type 4).

**FIGURE 5:**
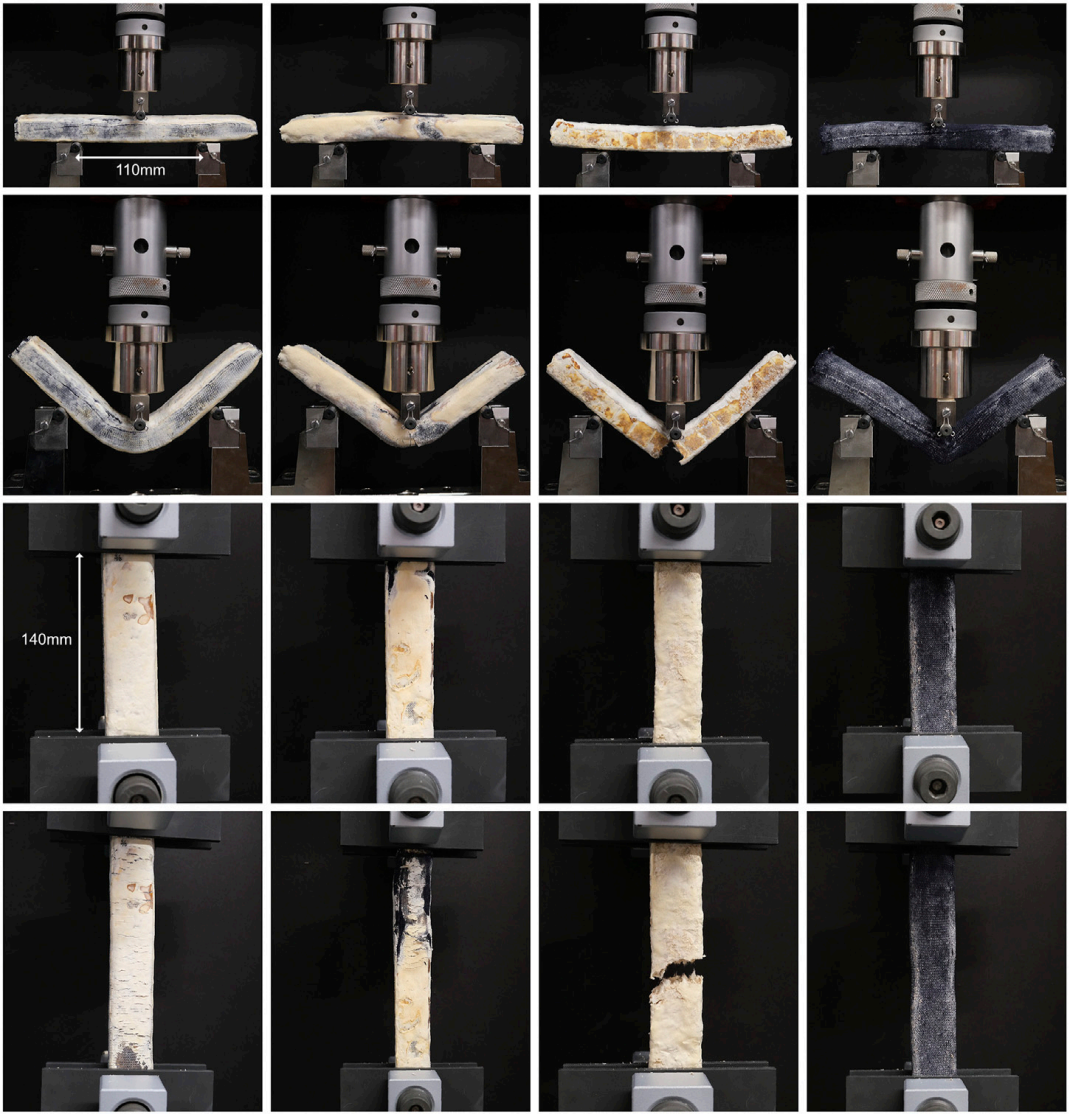
Specimen failure in bending and tension. Sample types 1 to 4 are shown from left to right. Sample type 3 (mycopaste with no textile formwork) exhibits brittle failure and cracks in both bending and tension; samples with textile formwork are held together by the textile tube.

To elucidate the impact of the knitted textile formwork on the mechanical properties and failure behaviour, sample type 3 was identical to type 2 but without the textile formwork. It was anticipated that the textile formwork would increase the strength of the samples by providing a fibrous reinforcement at the surface which would prevent cracking and therefore increase the tensile and flexural strength, and also provide restraint against splitting in compression. This was confirmed by the test results, which showed a reduction of between 14% and 50% in mean strength and Young’s modulus for all tests. Without the textile formwork, the type 3 samples cracked completely and lost all strength after failure (Figure 5). In contrast, the type 2 samples were held together by the formwork and continued to sustain load with an approximately horizontal stress-strain curve after failure (Figures 4, 5). This provides a potentially useful safety mechanism to prevent collapse of a structure after exceptional loading, similar to the use of highly ductile structural steel to prevent collapse.

The type 4 test specimens were made without mycelium to understand the impact of the mycelium growth on the material properties. Without mycelium the mean strength and Young’s modulus was reduced for all samples; by 50%–60% for the flexural tests, but only 9%–25% for the compression tests. Even with a 50%–60% reduction, the flexural strength and stiffness was still significantly higher than the type 1 “typical mycelium composite”. A possible explanation is that the paper powder and paper fibre clumps bond the substrate together as they dry, and this gives better mechanical performance than mycelium and sawdust alone. For some applications it may be worth considering using paper or other fine cellulose fibres as a bonding agent, eliminating the need to use mycelium. However, with mycelium in the mycocrete recipe (sample type 2) the strength and Young’s modulus are approximately double the values for sample type 4, giving excellent mechanical properties which are an order of magnitude higher than typical mycelium composites.

To understand the mechanical properties of mycocrete in the context of construction materials, we can make a comparison with hemp-lime concrete. Hemp-lime concrete, or “hempcrete”, is promoted as a carbon negative bulk construction material, however the production of the lime binder emits CO2, which is offset by carbon sequestration in the hemp. Without the need for extraction and processing of a mineral binder, mycelium materials have potential for even lower environmental impact. Tests on a range of different hemp-lime concrete mixes gave compressive strength values of 0.29–0.39 MPa and flexural strength of 0.1–0.2 MPa (Walker et al., 2014). The values for mycocrete (Table 4, sample type 2) are approximately 3x higher for compressive strength and 10x higher for flexural strength. Whilst providing superior properties to hempcrete, the mechanical properties of mycocrete are considerably lower than structural timber, e.g., C16 grade softwood, compression strength parallel to the grain = 17 MPa, perpendicular to the grain = 2.2 MPa (BS EN 338:2009, Structural timber. Strength classes). This positions mycocrete as a relatively high strength bio-based bulk construction material, which could be used in the form of self-supporting infill panels within a frame structure, with potential to be used as a load bearing structure for small buildings, subject to further testing.

### 4.3 Large scale application of mycocrete paste with knitted formwork

**FIGURE 6:**
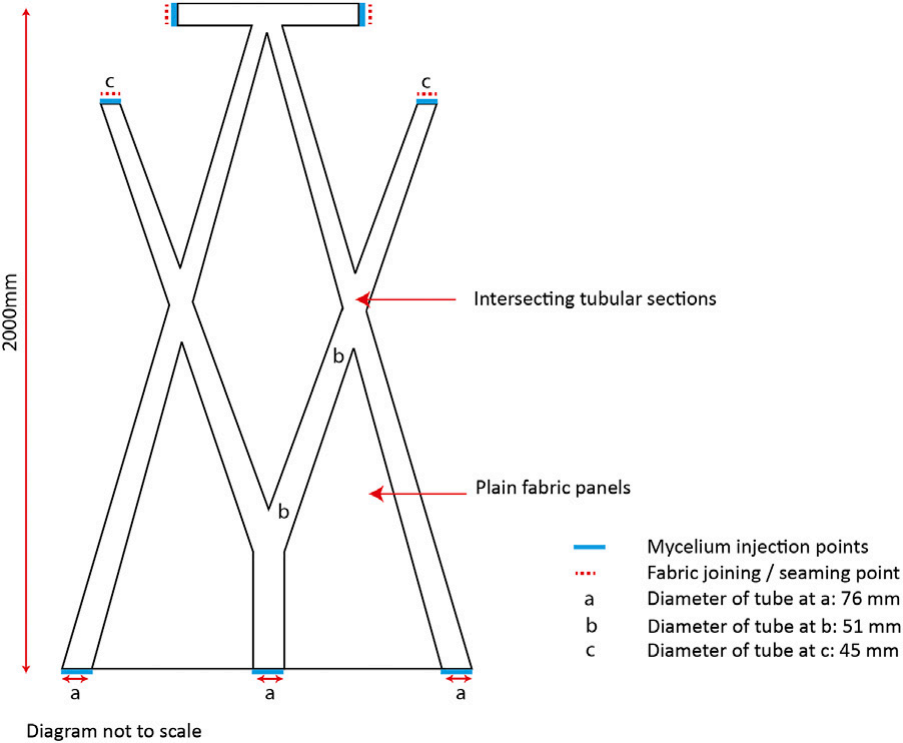
Dimensions and placement of intersecting tubular sections and seaming points within one knitted module for the BioKnit prototye.

To test mycocrete at scale the new paste formulation was used in combination with permanent knitted formwork in the biofabrication of the BioKnit prototype. BioKnit is composed of seven individual knitted modules each containing an internal system of integral interconnecting tubes. The modules are designed to be assembled into one preform (Figure 6). Each module is joined with four seams, and the tubular knitted sections form slender catenary arches that act as permanent textile formwork in the final grown structure (Agraviador et al., 2022).

To grow the BioKnit prototype three major components were required; the assembled knit preform, the mycocrete paste and an external framework that acted as the environmental growing chamber and integrated hanging system that enabled the catenary arch structure to be produced by suspending the whole structure during the growing period. Each component was prepared separately, including autoclaving the knit preform and substrates before everything was moved to site for the build. The paste recipe comprised two stages; firstly 5.6 kg mycelium spawn was pre-grown on 28 kg sawdust fibers saturated with 28 kg water, as detailed above. Secondly the paste was prepared to the correct proportions (Table 2). This was undertaken onsite and mixed as required during the build.

**FIGURE 7:**
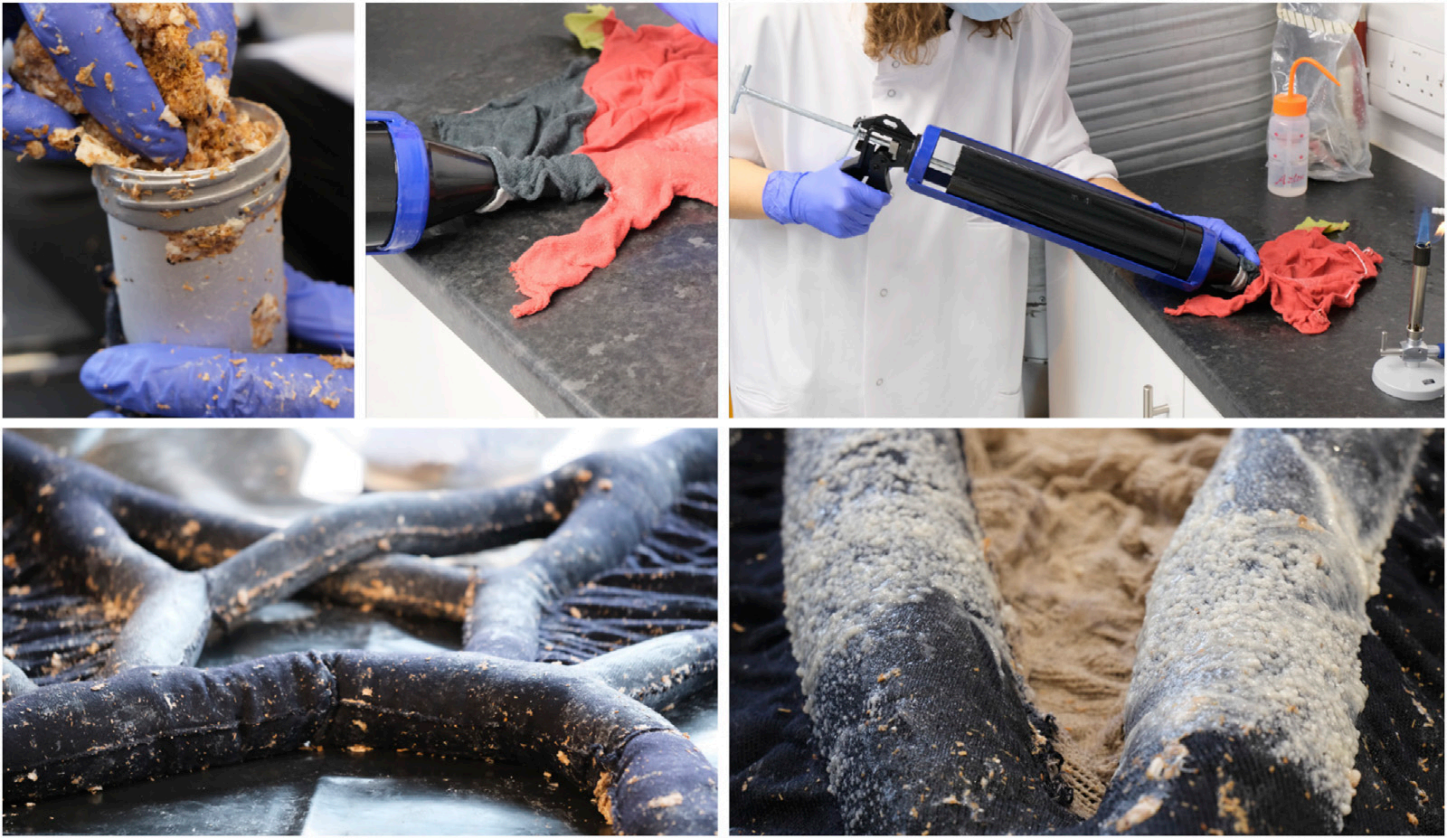
Paste injection process using extrusion gun. Illustration shows the paste consistency (top left), injection technique (top centre and right), and the filled tubular components at maximum capacity (bottom).

One challenge in moving from standard length tubular test samples to an architectural prototype was that the tube length increased to over 2000 mm in length whilst the tube diameter ranged from 45 to 76 mm. To produce a robust composite the tubes needed to be filled evenly, with the knitted preform fully expanded with mycelium. The system devised to fill the tubes comprised a manually filled injection gun with 17 mm nozzle. The injection gun was filled with the mycocrete paste, and then positioned so that the nozzle was inserted into the tubular fabric at one of several filling points positioned throughout the preform (Figure 7). Working on one module at a time, the paste was injected into the fabric at a consistent rate working from the middle outwards. The fabric was gathered up around the nozzle and as the paste was injected into the knit, the gun was pulled back to allow the fabric to expand with mycelium paste at a consistent rate. Once the interconnecting tubes were completely full, the filling points were hand sewn to close each seam.

### 4.4 Challenges for injection-based fabrication with mycocrete paste

To achieve a smooth injection process the paste consistency was critical. There were two key aspects to control here; the elimination of clumps that formed during pregrowth, and the adaptation of the additional water content required. Clumps can form during the pregrowth of mycelium and sawdust as the mycelium begins to bind to the substrate, and so the decision was taken to reduce the pregrowth stage to a maximum of 5 days. For this prototype the paste was mixed by hand so clumps that had formed needed to be broken down manually to avoid blocking the nozzle of the extrusion gun. In future work this process will be automated using industrial blenders so that clumps can be eliminated during the mixing of the paste. Water represented 75% of the total weight of the prototype during growth. In addition to saturating the textile preform the mycocrete paste contained approximately 75% water. It was found that time was a crucial parameter for the consistency of the mix, which varied with the amount of time that the paper based materials had been allowed to soak for prior to mixing. A shorter soaking time led to a smoother paste formulation, leading to enhanced injectability. The injection process was time consuming but has potential to be automated.

### 4.5 Opportunities for injection-based fabrication with permanent fabric formwork

This prototype demonstrates the opportunity to utilize the potential for lightweight construction and complex form offered by a textile formwork with low impact fungal-based biofabrication. BioKnit was grown in one piece rather than assembled from multiple pregrown sections. There are several advantages to this approach. Firstly, the structure is continuous with no connections which can be weak points in a structure and often require complex fabrication. In addition, this approach exploits the textiles’ lightweight formability and the structural efficiency of the catenary geometry. The use of flexible knitted formwork with viscous mycocrete paste facilitated the design of an efficient and lightweight structure that incorporated a complex pattern of interconnecting tubes that once filled produce the suspended catenary arch structure.

In the design stage the design of the fabric and the design of the paste mixture were developed concurrently through an iterative process. To allow for narrow diameters in the tubular knit formwork, a smooth viscous paste was required. To enable the length of the tubes to be filled, the injection system was developed. The flexibility of the injection process enabled different diameters of tube to be filled without the need for multiple filling points in the fabric. Since tube diameter is an important parameter for creating complex geometries, this technique opens up novel possibilities for creating alternative tubular configurations to produce stronger, self-supporting designs. For example, narrower tube diameters could be used to reduce the material volume required for arches to lower the overall material usage in the structure.

## 5 Conclusion

The findings of this paper show that use of permanent knitted formwork in combination with the novel mycocrete mycelium paste improves the performance of mycelium composites in comparison with samples containing both conventional sawdust substrates, and samples without textile formwork. Mechanical testing showed a dramatic increase in tensile, compressive, and flexural strength for samples that combine mycocrete paste with textile formwork. For example, the mean flexural modulus increased by a factor of 16 compared to tests on a typical sawdust based mycelium composite which was tested in identical conditions. This shows that there is potential to tailor mycelium composite recipes for specific applications, and potentially to achieve much better mechanical performance than is currently reported in literature. The ability to scale up the biofabrication process for use at an architecture scale was explored through discussion of the BioKnit prototype. The use of permanent integrated knitted formwork alongside mycocrete paste enabled the production of a self-supporting arched dome using a system of very slender tubular arches. The specific formulation of mycocrete was essential to achieve the correct consistency of mycelium paste to allow application with an injection gun. In turn, the use of the injection gun produced a consistent fill throughout the knitted formwork and resulted in excellent mycelium growth and integration with the textile formwork. This research demonstrates how biofabrication requires flexibility and adaptability in both fabrication processes and materials used. The integration of mycocrete paste with a controlled injection process used with permanent knitted formwork resulted in a robust production system for textile biohybrid composites with potential to be further developed and scaled for applications in construction.

## Data Availability

The raw data supporting the conclusion of this article will be made available by the authors, without undue reservation.
